# Newborn Screening for 6 Lysosomal Storage Disorders in China

**DOI:** 10.1001/jamanetworkopen.2024.10754

**Published:** 2024-05-13

**Authors:** Siyu Chang, Xia Zhan, Yuchao Liu, Huanlei Song, Zizhen Gong, Lianshu Han, Gustavo H. B. Maegawa, Xuefan Gu, Huiwen Zhang

**Affiliations:** 1 Department of Pediatric Endocrinology and Genetics, Xinhua Hospital, Shanghai Institute for Pediatric Research, Shanghai Jiao Tong University School of Medicine, Shanghai, China; 2 Department of Pediatrics, Columbia University Vagelos College of Physicians and Surgeons, New York, New York; 3 Columbia University Medical Center, New York, New York

## Abstract

**Importance:**

Newborn screening (NBS) for lysosomal storage disorders (LSDs) is becoming an increasing concern in public health. However, the birth prevalence of these disorders is rarely reported in the Chinese population, and subclinical forms of diseases among patients identified by NBS have not been evaluated.

**Objective:**

To evaluate the birth prevalence of the 6 LSDs in the Shanghai population and determine subclinical forms based on clinical, biochemical, and genetic characteristics.

**Design, Setting, and Participants:**

This cohort study included 50 108 newborns recruited from 41 hospitals in Shanghai between January and December 2021 who were screened for 6 LSDs using tandem mass spectrometry (MS/MS). Participants with screen-positive results underwent molecular and biochemical tests and clinical assessments. Data were analyzed from January 2021 through October 2022.

**Exposures:**

All participants were screened for Gaucher, acid sphingomyelinase deficiency (ASMD), Krabbe, mucopolysaccharidosis type I, Fabry, and Pompe diseases using dried blood spots.

**Main Outcomes and Measures:**

Primary outcomes were the birth prevalence and subclinical forms of the 6 LSDs in the Shanghai population. Disease biomarker measurements, genetic testing, and clinical analysis were used to assess clinical forms of LSDs screened.

**Results:**

Among 50 108 newborns (26 036 male [52.0%]; mean [SD] gestational age, 38.8 [1.6] weeks), the mean (SD) birth weight was 3257 (487) g. The MS/MS-based NBS identified 353 newborns who were positive. Of these, 27 newborns (7.7%) were diagnosed with 1 of 6 LSDs screened, including 2 newborns with Gaucher, 5 newborns with ASMD, 9 newborns with Krabbe, 8 newborns with Fabry, and 3 newborns with Pompe disease. The combined birth prevalence of LSDs in Shanghai was 1 diagnosis in 1856 live births, with Krabbe disease the most common (1 diagnosis/5568 live births), followed by Fabry disease (1 diagnosis/6264 live births), and ASMD (1 diagnosis/10 022 live births). Biochemical, molecular, and clinical analysis showed that early-onset clinical forms accounted for 3 newborns with positive results (11.1%), while later-onset forms represented nearly 90% of diagnoses (24 newborns [88.9%]).

**Conclusions and Relevance:**

In this study, the combined birth prevalence of the 6 LSDs in Shanghai was remarkably high. MS/MS-based newborn screening, combined with biochemical and molecular genetic analysis, successfully identified and characterized newborns who were screen-positive, which may assist with parental counseling and management decisions.

## Key Points

**Question:**

What are the birth prevalence and subclinical forms of the 6 lysosomal storage disorders (LSDs) in the Shanghai population?

**Findings:**

In this cohort study of 50 108 newborns in Shanghai, tandem mass spectrometry (MS/MS)–based newborn screening for 6 LSDs identified 353 newborns who were positive. Further molecular, biochemical, and clinical analysis confirmed 27 of these newborns (1 in 1856 live births), among whom 3 newborns (11.1%) had early-onset clinical forms and 24 newborns (88.9%) had later-onset forms.

**Meaning:**

These findings suggest that expanded newborn screening for LSDs in China is warranted; the high birth prevalence and clinical subtype ascertainment support the application of MS/MS to serve as a first-tier screening approach.

## Teaser

This cohort study investigates prevalence of 6 lysosomal storage disorders and their subclinical forms among newborns in Shanghai, China.

## Introduction

Lysosomal storage disorders (LSDs) make up a group of inborn organelle disorders caused by the deficiency of specific lysosomal enzymes or transport proteins, resulting in increased accumulation of macromolecules and lysosomal dysfunction.^1,2^ LSDs comprise nearly 60 distinct disease entities, most of which have autosomal recessive inheritance but a few of which have X-linked inheritance, including Fabry disease and mucopolysaccharidosis type II.^3,4^ The combined incidence of LSDs is estimated to be 1 in 4000 to 1 in 9000 live births according to a 2022 report by the American College of Medical Genetics and Genomics (ACMG).^5^ LSDs can affect multiple organs, especially the central nervous system, and patients who are untreated may be at risk of lifelong disability or even death.^6^ Treatment options for LSDs include enzyme replacement therapy, hematopoietic stem cell transplantation, and substrate reduction therapy.^7,8^ Early diagnosis and treatment initiation are essential for reducing disease-related morbidity and mortality.

Some countries have included some LSDs in their newborn screening (NBS) programs.^9,10,11^ Using total blood specimens collected on dried blood spot (DBS) cards, NBS assays for LSDs are performed as enzyme activity–based analyses and divided into 2 types depending on the readout of the specific enzyme product: tandem mass spectrometry (MS/MS) and synthetic and fluorescent-based substrate.^12,13^ The advantage of the MS/MS is that it can simultaneously measure enzymatic activities of 6 lysosomal hydrolases, and it has gradually become the preferred large-scale NBS assay for these LSDs.^10^ To our knowledge, only 1 pilot study for LSDs in Shandong province, mainland China, has been carried out.^14^ However, the study did not distinguish between clinical forms of LSDs screened, which is critical for subsequent genetic counseling and management decisions. Additionally, more data need to be accumulated to investigate the birth prevalence of LSDs in China.

To address these gaps, we conducted a pilot NBS study for 6 LSDs, including Gaucher, acid sphingomyelinase deficiency (ASMD), Krabbe, mucopolysaccharidosis type I (MPS-I), Fabry, and Pompe diseases, using MS/MS assay in DBS specimens collected from live births in the major hospitals of Shanghai, China. We identified 27 patients from 50 108 newborns and performed biochemical and molecular genetics confirmatory tests and clinical assessments. We aimed to provide a more accurate estimated prevalence of 6 LSDs and their subclinical forms in Shanghai.

## Methods

The Ethical Committee of Xinhua Hospital, Shanghai Jiaotong University School of Medicine, approved this cohort study. Written informed consent was obtained from parents or guardians of participants. This study followed the Strengthening the Reporting of Observational Studies in Epidemiology (STROBE) reporting guideline.

### Study Design and Participant

The Xinhua Hospital, Shanghai Jiaotong University School of Medicine, was the first center to introduce the NBS for 6 LSDs in the Shanghai region. From January to December 2021, a total of 50 108 DBS samples from live newborns were collected from 41 maternity hospitals in Shanghai within 72 hours to 7 days after birth. DBS samples were sent to our center by express delivery for testing. Newborns with positive results in the initial screening and reexamination of the original DBS specimen underwent genetic testing. Those with positive genetic results underwent biomarker testing and clinical evaluation. Inherited metabolic disease specialists performed diagnosis and subtype confirmation.

### Lysosomal Enzyme Activity Assay

The NeoLSD MSMS Kit assay system (PerkinElmer) was used to measure enzyme activities of acid-β-glucocerebrosidase (ABG; for Gaucher disease), ASM (for ASMD), β-galactocerebrosidase (GALC; for Krabbe disease), α-L-iduronidase (IDUA; for MPS-I), α-galactosidase A (GLA; for Fabry disease), and acid-α-glucosidase (GAA; for Pompe disease). DBS samples and quality controls (3.2 mm) were punched into 96-well plates, with 30 μL per well of incubation cocktail added, then incubated for 18 hours in the shaking incubator (MB100-2A; Thermo) at 37 °C and 400 rpm. The reaction was terminated by adding 100 μL per well of the quench solution. After being pipetted up and down 10 times, the contents of each well were transferred to a deep-well plate. Liquid-liquid extraction was performed by adding 400 μL of NeoLSD Extraction Solution and 200 μL of water, then mixing the contents with a pipette (20 times). The organic top layer (50 μL) was transferred into a new 96-well plate after centrifuging for 5 minutes at 700 g, dried with nitrogen, and resuspended with 100 μL of flow solvent. Flow injection analysis–MS/MS was used to measure internal standards and enzyme-generated products using multiple reaction monitoring. Parameters of the liquid tandem mass spectrometer (QSight 210 MD; PerkinElmer) are shown in eTable 1 in Supplement 1. The mean blank value on each plate was subtracted from each enzyme activity to obtain activity values of samples.

### Gene Variant Analysis

Except for samples with GAA activity greater than 10% of the daily median, genetic analysis was performed on newborns with positive initial screening and reexamination results. Genomic DNA of newborns who were presumed positive and their parents was extracted from initial screening DBS samples or peripheral venous blood samples using a DNA extraction kit (Hieff NGS OnePot DNA Library Prep Kit for Illumina; Yeasen). Target gene exons and adjacent spliced regions (approximately 20 base pair before or after) were captured and enriched by probe hybridization. Target next-generation sequencing was carried out on newborns with suspected disease using a genetic diagnosis panel of inherited metabolic disorders covering 132 diseases, 102 genes, and 15 mitochondrial genomes. Identified variants were nominated using human *GBA* (NM_001005741), *SMPD1* (NM_000543.5), *GALC* (NM_000153.4), *IDUA* (NM_000203.5), *GLA* (NM_000169.3), and *GAA* (NM_000152.5) sequences as references. Novel variants were classified according to ACMG standards and guidelines.^15^

### Biochemical Marker Assays of DBS

Assessed biochemical markers included glucosylsphingosine for Gaucher disease, psychosine (psy) for Krabbe disease, and globotriaosylsphingosine (lyso-Gb3) for Fabry disease. These were detected in identified patients using previously reported methods.^16,17,18^

### Statistical Analysis

The cutoff value of the 6 enzyme activities was set at 20% of the median value of each test based on the previous report.^2^ We used 3 quality controls with different concentrations (low, medium, and high) provided by PerkinElmer, which were added to each plate to ensure the day-to-day validity of results. Mass spectrometry data were analyzed using Simplicity 3Q MD software (PerkinElmer). Statistical analysis was performed using SPSS statistical software version 16.0 (IBM). Data were analyzed from January 2021 through October 2022.

## Results

**Figure 1.**
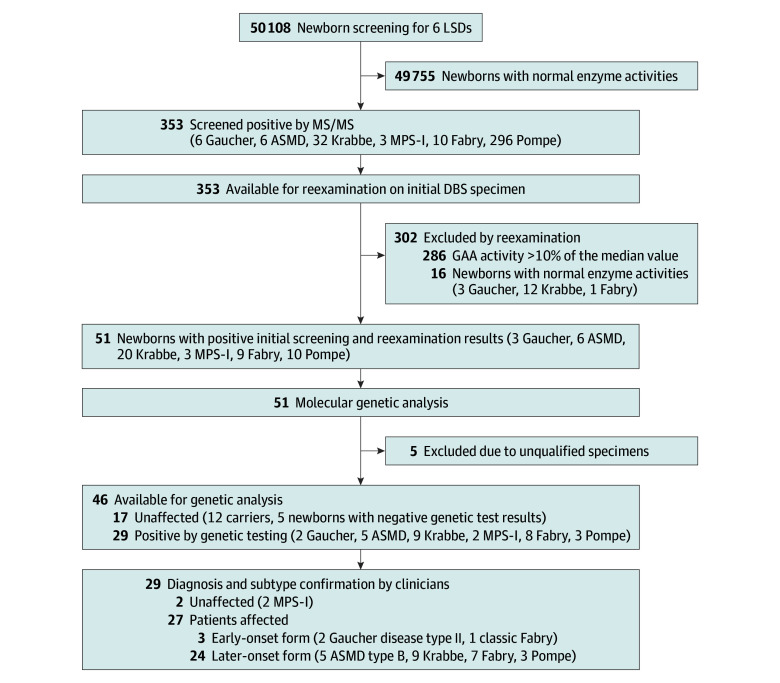
Flow Diagram for LSD Screening ASMD indicates acid sphingomyelinase deficiency; DBS, dried blood spot; GAA, α-glucosidase; LSD, lysosomal storage disease; MPS-I, mucopolysaccharidosis type I; MS/MS, tandem mass spectrometry.

**Figure 2.**
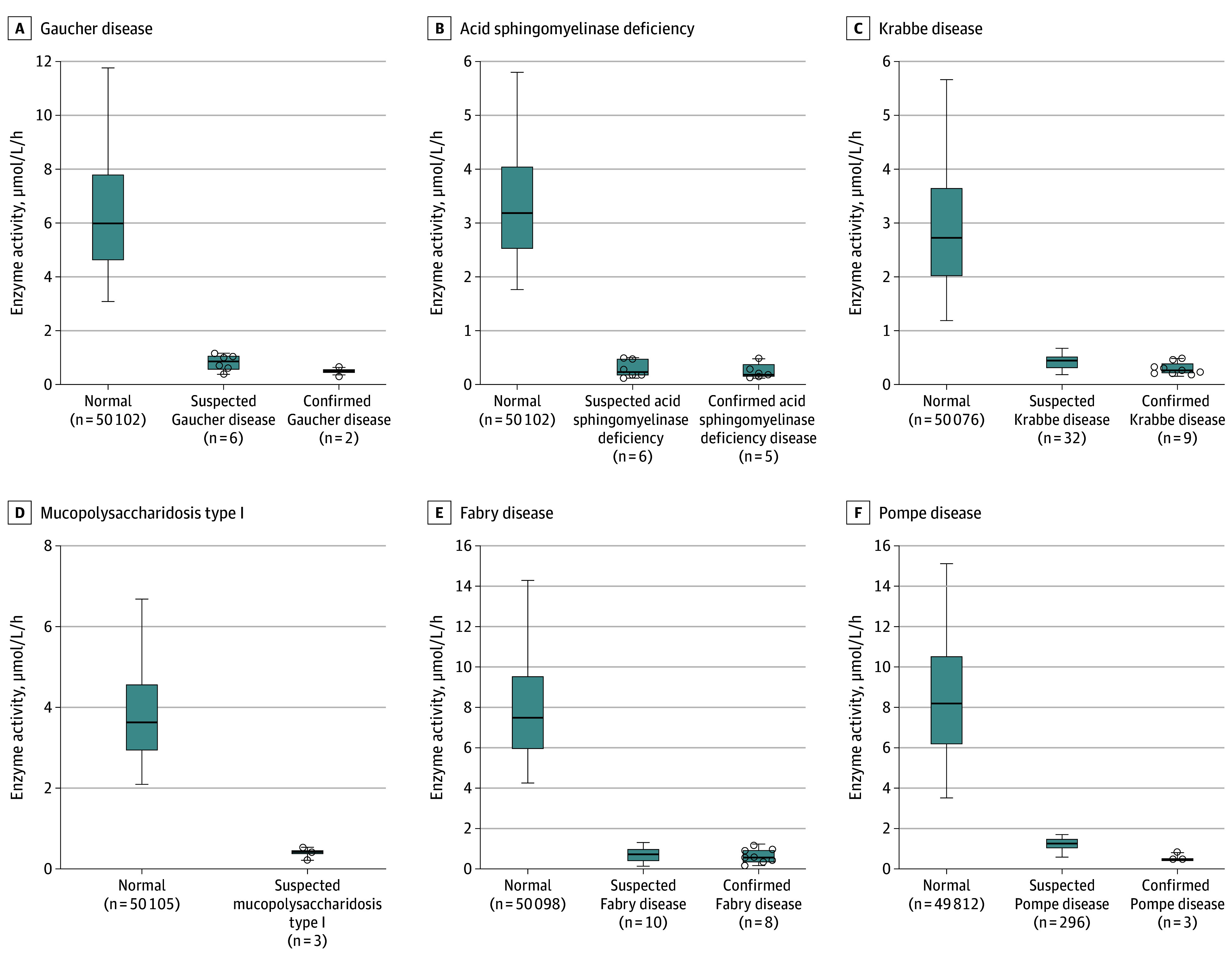
Enzyme Activity Levels Enzyme activity levels of 6 lysosomal storage diseases are shown in newborns with normal levels, those with suspected disease (decreased enzyme activity), and those with confirmed disease. Whiskers indicate minimum and maximum ranges for categories with fewer than 10 samples, while 5th to 95th percentiles are indicated for those with more than 10 samples. Symbols in categories with fewer than 10 samples indicate raw enzyme activity level. The line inside the box indicates the median value.

Among 50 108 screened newborns (26 036 male [52.0%]; mean [SD] gestational age, 38.8 [1.6] weeks), the mean (SD) birth weight was 3257 (487) g. There were 353 infants identified with low levels of enzyme activities (positive rate, 0.7%), including 6 infants with enzymes associated with Gaucher, 6 infants with enzymes associated with ASMD, 32 infants with enzymes associated with Krabbe, 3 infants with enzymes associated with MPS-I, 10 infants with enzymes associated with Fabry, and 296 infants with enzymes associated with Pompe disease (Table 1). Among 51 newborns with positive initial screening and reexamination results, 5 newborns were excluded owing to unqualified specimens (9.8%) and 46 newborns (90.2%) underwent genetic analysis, of which 29 newborns (63.0%) were positive (Figure 1). After further biomarker testing and clinical evaluation by metabolic disease specialists, 27 newborns (7.7% of all infants with low enzyme activity) were diagnosed with LSDs, consisting of 2 newborns with Gaucher, 5 newborns with ASMD, 9 newborns with Krabbe, 8 newborns with Fabry, and 3 newborns with Pompe disease (Table 1). The 8 infants with Fabry disease were all male (0.03% or 1 in 3300 among all males). The overall birth prevalence of LSDs in Shanghai was 1 diagnosis in 1856 live births, while the birth prevalences of Gaucher, ASMD, Krabbe, Fabry, and Pompe disease were 1 diagnosis in 25 054 live births, 10 022 live births, 5568 live births, 6264 live births, and 16 702 live births, respectively (eTable 3 in Supplement 1; Table 1). The mean (SD) enzyme activity levels of ABG (0.49 [0.17] μmol/L/h), ASM (0.26 [0.13] μmol/L/h), GALC (0.27 [0.10] μmol/L/h), GLA (0.64 [0.34] μmol/L/h), and GAA (0.56 [0.20] μmol/L/h) in confirmed diagnoses were lower than those in newborns who were screened negative (ABG: 6.56 [3.10] μmol/L/h; ASM: 3.43 [1.43] μmol/L/h; GALC: 3.01 [1.63] μmol/L/h; GLA: 8.21 [3.52] μmol/L/h; GAA: 8.53 [3.65] μmol/L/h) (Figure 2).

**Table 1.** NBS Results for 6 LSDs

Parameter	Newborns, No.						
	Gaucher disease	ASMD	Krabbe disease	MPS-I	Fabry disease	Pompe disease	Total
Positive initial NBS	6	6	32	3	10	296	353
Confirmed LSD	2	5	9	0	8	3	27
Carrier	1	1	9	0	0	1	12
PPV (95% CI), %^a^	33.33 (6.00-75.89)	83.33 (36.48-99.12)	28.13 (14.40-46.98)	0 (0-12.01)	80.0 (44.22-96.4)	1.01 (0.26-3.18)	7.65 (5.19-11.06)
Prevalence, No. diagnoses:No. live births	1:25 054	1:10 022	1:5568	NA	1:6264	1:16 702	1:1856

Abbreviations: ASMD, acid sphingomyelinase deficiency; LSD, lysosomal storage disease; MPS-I, mucopolysaccharidosis type I; NA, not applicable; NBS, newborn screening; PPV, positive predictive value.

^a^ PPV is calculated as true positives divided by test positive outcomes.

For Gaucher, 6 newborns displayed ABG activity below 20% of the median, and 2 newborns were confirmed to have *GBA* gene pathogenic variants by genetic analysis. Patient 1 had compound heterozygosity, with pathogenic variants of c.1448T>C (p.Leu483Pro) and c.[1448T>C;1483G>C;1497G>C] (p.Leu483Pro; Ala495Pro; Val499Val). Patient 2 had 2 pathogenic variants: c.1448T>C (p.Leu483Pro) and c.611dup (p.Gln205Alafs*57) (Table 2). The p.Leu483Pro variant was a pathogenic variant associated with neurological alterations.^16^ The mean (SD) leukocyte ABG activity level in these 2 patients was 1.54 (0.99) nmol/h/mg protein (cutoff value, 6.56-55.10 nmol/h/mg protein). Their mean (SD) glucosylsphingosine concentration in DBS samples (173.5 [44.29] ng/mL; cutoff value <10.0 ng/mL) was substantially increased. Based on these results, these patients were categorized as having Gaucher disease type II, which was the most severe form of neurological involvement of Gaucher disease.

**Table 2.** Biochemical, Genetic, and Clinical Analyses of Patients With Lysosomal Storage Diseases

Patient	Sex	Enzyme activity		Disease biomarker level^c^	Gene	Variant		ACMG classification	Phenotype
		In DBS, μmol/L/h^a^	In white cell, nmol/h/mg protein^b^			Nucleotide	Protein		
**Gaucher disease (ABG)**									
P1	F	0.37	2.24	204.8 ng/mL lyso-Gb1	*GBA*	c.1448T>C c.[1448T>C; 1483G>C;1497G>C]	p.Leu483Pro p.Leu483Pro; Ala495Pro;Val499Val	Pathogenic Pathogenic	Gaucher type II
P2	M	0.61	0.84	142.2 ng/mL lyso-Gb1	*GBA*	c.1448T>C c.611dup	p.Leu483Pro p.Gln205Alafs*57	Pathogenic Pathogenic	Gaucher type II
**ASMD (ASM)**									
P1	M	0.41	NA	NA	*SMPD1*	c.689G>A c.995C>G	p.Arg230His p.Pro332Arg	LP LP	ASMD type B
P2	M	0.39	NA	NA	*SMPD1*	c.1598C>T c.1805G>A	p.Pro533Leu p.Arg602His	VUS Pathogenic	ASMD type B
P3	M	0.19	NA	NA	*SMPD1*	c.995C>G c.995C>G	p.Pro332Arg p.Pro332Arg	LP LP	ASMD type B
P4	F	0.13	NA	NA	*SMPD1*	c.995C>G c.995C>G	p.Pro332Arg p.Pro332Arg	LP LP	ASMD type B
P5	M	0.19	3.72	NA	*SMPD1*	c.995C>G c.995C>G	p.Pro332Arg p.Pro332Arg	LP LP	ASMD type B
**Krabbe disease (GALC)**									
P1	F	0.24	NA	1.41 nmol/L psy	*GALC*	c.1901T>C c.1901T>C	p.Leu634Ser p.Leu634Ser	Pathogenic Pathogenic	Later onset
P2	M	0.31	4.12	NA	*GALC*	c.1901T>C c.1901T>C	p.Leu634Ser p.Leu634Ser	Pathogenic Pathogenic	Later onset
P3	M	0.22	NA	0.87 nmol/L psy	*GALC*	c.1901T>C c.1861C>T	p.Leu634Ser p.Arg621Cys	Pathogenic VUS	Later onset
P4	M	0.24	11.51	1.56 nmol/L psy	*GALC*	c.1901T>C c.1901T>C	p.Leu634Ser p.Leu634Ser	Pathogenic Pathogenic	Later onset
P5	F	0.27	NA	4.17 nmol/L psy	*GALC*	c.1901T>C c.1901T>C	p.Leu634Ser p.Leu634Ser	Pathogenic Pathogenic	Later onset
P6	M	0.33	NA	0.39 nmol/L psy	*GALC*	c.1901T>C c.1042A>T	p.Leu634Ser p.Thr348Ser	Pathogenic VUS	Later onset
P7	M	0.17	NA	6.79 nmol/L psy	*GALC*	c.1901T>C c.461C>A	p.Leu634Ser p.Pro154His	Pathogenic LP	Later onset
P8	F	0.18	NA	0.28 nmol/L psy	*GALC*	c.1901T>C c.1901T>C	p.Leu634Ser p.Leu634Ser	Pathogenic Pathogenic	Later onset
P9	F	0.49	NA	1.71 nmol/L psy	*GALC*	c.1901T>C c.1901T>C	p.Leu634Ser p.Leu634Ser	Pathogenic Pathogenic	Later onset
**Fabry disease (GLA)**									
P1	M	0.41	1.07	12.96 nmol/L lyso-Gb3	*GLA*	c.718_719del	p.Lys240Glufs*9	Pathogenic	Classic
P2	M	1.17	5.89	0.62 nmol/L lyso-Gb3	*GLA*	c.640-801G>A	NA	Pathogenic	Later onset
P3	M	0.71	7.61	1.77 nmol/L lyso-Gb3	*GLA*	c.428C>T	p.Ala143Val	VUS	Later onset
P4	M	0.45	3.98	0.74 nmol/L lyso-Gb3	*GLA*	c.358C>G	p.Leu120Val	LP	Later onset
P5	M	0.18	1.05	0.51 nmol/L lyso-Gb3	*GLA*	c.335G>A	p.Arg112His	LP	Later onset
P6	M	0.97	NA	0.43 nmol/L lyso-Gb3	*GLA*	c.1067G>A	p.Arg356Gln	Pathogenic	Later onset
P7	M	0.62	NA	0.56 nmol/L lyso-Gb3	*GLA*	c.593T>C	p.Ile198Thr	Pathogenic	Later onset
P8	M	0.55	NA	0.73 nmol/L lyso-Gb3	*GLA*	c.137A>C	p.His46Pro	LP	Later onset
**Pompe disease (GAA)**									
P1	F	0.71	NA	NA	*GAA*	c.2189 + 22G>A c.2238G>C	NA p.Trp746Cys	VUS Pathogenic	Later onset
P2	M	0.45	NA	NA	*GAA*	c.2238G>C c.2662G>T	p.Trp746Cys p.Glu888*	Pathogenic Pathogenic	Later onset
P3	M	0.49	NA	NA	*GAA*	c.1622C>T c.2238G>C	p.Pro541Leu p.Trp746Cys	VUS Pathogenic	Later onset

Abbreviations: ABG, acid-β-glucocerebrosidase; ACMG, American College of Medical Genetics and Genomics; ASM, acid sphingomyelinase; ASMD, acid sphingomyelinase deficiency; DBS, dried blood spot; F, female; GAA, α-glucosidase; GALC, β-galactocerebrosidase; GLA, α-galactosidase; LP, likely pathogenic; lyso-Gb1, glucosylsphingosine; lyso-Gb3, globotriaosylsphingosine; M, male; NA, not available; psy, psychosine; VUS, variant of uncertain significance.

^a^ Cutoff value of ABG, ASM, GALC, GLA, and GAA enzyme activities in DBS: 20% of the median value of each test (mean [SD] cutoff value, 1.23 [0.25] μmol/L/h, 0.64 [0.11] μmol/L/h, 0.55 [0.10] μmol/L/h, 1.52 [0.17] μmol/L/h, and 1.63 [0.23] μmol/L/h, respectively).

^b^ Cutoff value of ABG, ASM, GALC, and GLA in white cell: 6.56 to 55.10 nmol/h/mg protein, 12.02 to 114.50 nmol/17h/mg protein, 12.89 to 100.93 nmol/17h/mg protein, and 16.04 to 145.5 nmol/h/mg protein, respectively.

^c^ Cutoff value of disease biomarkers: lyso-Gb1 (Gaucher), less than 10 ng/mL; psy (Krabbe), less than 0.39 nmol/L; lyso-Gb3 (Fabry), less than 1.11 nmol/L.

Molecular analysis for ASMD revealed that 5 of 6 newborns who screened positive had *SMPD1* variants. Patient 1 had compound heterozygosity, with pathogenic variants of c.689G>A (p.Arg230His) and c.995C>G (p.Pro332Arg). Patient 2 had missense variants c.1598C>T (p.Pro533Leu) and c.1805G>A (p.Arg602His). Patients 3, 4, and 5 had the same homozygous variant of c.995C>G (p.Pro332Arg) (Table 2). The p.Arg602His variant occurred once, although it has been reported as a common variant in the Chinese clinical ASMD type B population.^19^ The p.Pro332Arg variant occurred in 4 individuals and was the most common variant, with a frequency of 7 of 10 alleles (70.0%). The p.Pro332Arg variant has been reported as a pathogenic variant, and its homozygosity was associated with a mild form of ASMD type B adult.^20^ Based on these results, these 5 patients were classified as having ASMD type B.

Initially, 32 newborns presented GALC activity of less than 20% of the median. The molecular genetic analysis confirmed this finding in 9 of them. Thus, Krabbe disease was the most commonly occurring disease, accounting for 9 of 27 LSDs (33.3%). Patients 1, 2, 4, 5, 8, and 9 had the same homozygous *GALC* variant of c.1901T>C (p.Leu634Ser). Patients 3, 6, and 7 had compound heterozygosity, with variants of c.1901T>C (p.Leu634Ser) and other missense variants (Table 2). We identified 4 variants in the *GALC* gene from 18 variant alleles. Among them, the p.Leu634Ser variant was the most common, with a frequency of 15 alleles (83.3%), which has been reported to be associated with the later-onset form and mild adult phenotype.^21^ The p.Pro154His variant occurred in 1 of 9 diagnoses and is known to be the most common variant clinically in Chinese patients.^17^ The remaining 2 variants were novel and previously unreported. As mentioned in the literature, psy assay has emerged as a second-tier test, which can be used as a confirmatory assay in newborns screening positive as GALC deficient and can classify patients as having early-onset or later-onset Krabbe disease.^22^ Infants with low GALC activity levels who displayed intermediate psy values of 2 to 10 nmol/L were categorized at risk for later-onset Krabbe, while a psy value of 10 nmol/L or greater was consistent with the early-onset form.^22^ Psy levels in patients 1, 3, 4, 6, 8, and 9 (1.41, 0.87, 1.56, 0.39, 0.28, and 1.71 nmol/L, respectively) were less than 2 nmol/L at 3 days after birth, while concentrations in patient 5 and 7 (4.17 and 6.79 nmol/L, respectively) were between 2 and 10 nmol/L. Based on these data, these 9 patients were classified as at risk for later-onset Krabbe disease.

NBS for MPS-I disease identified 3 newborns with IDUA activity below 20% of the median value. The *IDUA* gene variant analysis found that newborn 1 presented c.164C>A (p.Pro55Gln), c.1093C>G (p.Leu365Val), and c.1828 + 5G>A variants, while newborn 2 had no detected variants. Newborn 3 presented c.355G>T (p.Asp119Tyr) and c.911del (p.Val304Glyfs*13) variants. The leukocyte IDUA activity in newborn 1 was 14.91 nmol/h/mg protein (cutoff value, 12.17-277.51 nmol/h/mg protein). The first morning–voided urinary glycosaminoglycan qualitative analysis in newborns 1 and 3 was negative, and their electrophoresis determination showed no heparan sulfate or dermatan sulfate bands. Therefore, these 3 newborns were false positive for MPS-I disease.

There were 10 newborns who displayed GLA enzyme activity at less than 20% of the median value for X-linked Fabry disease. Among them, the molecular genetic analysis confirmed 8 male newborns to have *GLA* variants. Patient 1 had a pathogenic frameshift variant, c.718_719del (p.Lys240Glufs*9), which was reported in a patient with a classic phenotype.^23^ Patient 2 had the later-onset c.640-801G>A variant (also described as IVS4 + 919G>A), a pathogenic variant associated with later-onset cardiac manifestations (only this 1 patient had this variant [12.5% of patients with *GLA* variants]).^24^ Patient 3 had a novel variant, c.428C>T (p.Ala143Val). Patients 4, 5, 6, 7, and 8 had previously reported pathogenic later-onset variants c.358C>G (p.Leu120Val), c.335G>A (p.Arg112His), c.1067G>A (p.Arg356Gln), c.593T>C (p.Ile198Thr), and c.137A>C (p.His46Pro) (Table 2).^23,25,26^ The median (range) age at diagnosis of patients reported with these variants reached 27 (0-64) years (eTable 2 in Supplement 1). The lyso-Gb3 level in DBS in patient 1 was substantially increased, 12 times higher than the upper limit of the normal range during the neonatal period, and it increased to 24 times at 4 months (12.96 and 26.83 nmol/L; cutoff value of lyso-Gb3 < 1.11 nmol/L). Patients 2, 4, 5, 6, 7, and 8 presented normal lyso-Gb3 levels (0.62, 0.74, 0.51, 0.43, 0.56, and 0.73 nmol/L, respectively) at 3 days after birth. The lyso-Gb3 value in patient 3 was 1.77 nmol/L at 3 days after birth, and the value increased to 4.43 nmol/L when this individual was recalled. The lyso-Gb3 concentration increased in patients 2 and 4, who were followed up at 10 and 6 months, with values of 3.42 and 2.73 nmol/L, respectively. These results suggest that patient 1 should be categorized as having classic Fabry disease (12.5%), while the remaining 7 patients had later-onset forms (87.5%).

NBS for Pompe disease revealed that 296 newborns displayed GAA activity levels at less than 20% of the daily median, yielding a positive rate of 0.59%. This high positive rate has primarily been associated with *GAA* pseudodeficiency variants, such as c.[1726G>A; 2065G>A], which occurred commonly in Asian populations.^27^ Previous studies indicated that 4 of 71 (5.6%) of newborns who were screened positive were diagnosed with Pompe disease in Japan; this rate was 9 of 104 newborns (8.7%) in Taiwan and 3 of 279 newborns (1.1%) in Shandong.^14,27^ The GAA activity level in 3 newborns in Shandong identified with Pompe disease was less than 6% of the daily median.^14^ Additionally, the GAA activity level in 5 patients in our hospital with clinical disease was approximately 5% of the daily median. Based on these results, we did not perform genetic testing for newborns with GAA activity between 10% and 20% of the median value at initial screening and reexamination, while we conducted genetic testing for newborns with enzyme activity levels at less than 10%. Genetic analysis revealed that the 3 patients exhibited the common *GAA* pathogenic variant of c.2238G>C (p.Trp746Cys) (Table 2), which is prevalent among Chinese patients with later-onset Pompe disease.^28^ These 3 patients were classified as having later-onset Pompe disease.

Overall, among 27 infants with a clinically confirmed LSD, 3 infants (11.1%) had early-onset forms and 24 infants (88.9%) had later-onset forms. Patients with early-onset forms included 2 infants with Gaucher disease type II and 1 infant with classic Fabry, while those with later-onset forms included 9 patients with Krabbe, 7 patients with Fabry, 5 patients with ASMD type B, and 3 patients with Pompe disease.

## Discussion

To our knowledge, this cohort study reports the largest NBS for 6 LSDs using the MS/MS method in mainland China. We successfully identified 27 patients with LSDs from 50 108 newborns, consisting of 2 newborns with Gaucher, 5 newborns with ASMD, 9 newborns with Krabbe, 8 newborns with Fabry, and 3 newborns with Pompe. We conducted a detailed analysis of the most prevalent pathogenic variants in the 6 LSDs screened. Furthermore, we classified patients’ early or later clinical forms based on clinical, molecular genetic, and biomarker evaluations. Our findings provide insights into the early identification and diagnosis of LSDs. More importantly, ascertainment of prevalence and subtypes support the utility of MS/MS as a first-tier screening tool for LSDs.

NBS in our cohort revealed an unexpectedly high overall birth prevalence of LSDs in the Chinese population, with a frequency of approximately 54 diagnoses per 100 000 live births, which was higher than the previously reported worldwide incidence of 7.6 to 25 diagnoses per 100 000 live births.^29^ Individually, the prevalence of disorders varies by region. Krabbe disease was the most common LSD in Shanghai, with a high prevalence of 18 diagnoses per 100 000 live births, which is consistent with findings in Sweden and the Czech Republic.^29^ Fabry was the second most common LSD in Shanghai, with a prevalence of 1 diagnosis in 6264 live births, lower than that reported in Austria (1 diagnosis in 3859 live births) but similar to that in Japan (1 diagnosis in 7057 live births).^30^ We found that ASMD was far more prevalent in Shanghai (1 diagnosis in 10 022 live births) than in Washington State, where the prevalence was approximately 1 diagnosis in 44 000 live births.^9^ For Pompe, the prevalence was 1 diagnosis in 16 702 live births, similar to that reported in the US (1 diagnosis in 17 731 live births).^31^ The incidence of Gaucher disease in this study (1 diagnosis in 25 054 newborns) was higher than the worldwide incidence (1 diagnosis per 100 000 live births) but lower than that in the Ashkenazi Jewish population (1 diagnosis per 450 births).^5^ However, we did not identify any patient with MPS-I disease, which has a high incidence in Washington State and Taiwan.^32^

As of June 2011, 62% of patients with Krabbe disease who were enrolled in the World-Wide Krabbe Registry manifested early-onset infantile form.^33^ However, the 8 years of experience in the NBS for Krabbe disease reported in New York State in 2016 revealed that 5 of 51 infants at risk of Krabbe were confirmed as having the early-onset form.^34^ Notably, our study found that later-onset forms accounted for 100% of patients with Krabbe disease. This is ascribed to the frequently occurring *GALC* p.Leu634Ser variant, which is associated with adult patients who were homozygous.^35^ The population frequency of the p.Leu634Ser variant in East Asian populations in the gnomAD database was 0.008. Therefore, the estimated incidence of homozygosity with the p.Leu634Ser variant in East Asia was 1 in 62 500 individuals. However, based on our genetic results of 6 homozygotes in 50 108 newborns, the population carrier rate of the p.Leu634Ser variant in east China is 1 in 46 individuals, which is exceptionally high. In addition, the age at onset of 1 reported patient with homozygous p.Leu634Ser variant reached 50 years.^35^ This further indicates that the number of individuals at risk for Krabbe disease in the Chinese population may be high.

So far, NBS for Fabry disease has yielded a high incidence, with the predominance of *GLA* variants associated with later-onset clinical forms.^11,25^ In Taiwan, the frequency of Fabry was as high as 1 in 1 250 males, with 86% of patients having the later-onset IVS4 + 919G>A variant.^25^ Our study identified 8 patients with Fabry, 87.5% of whom had later-onset forms and 12.5% of whom had classic clinical forms. All patients with Fabry were males, for a mean of 1 in 3300 males. No specific *GLA* variant was identified in Fabry disease in mainland China. The IVS4 + 919G>A variant was uncommon, accounting for 12.5% of patients.

To our knowledge, this study is the first to evaluate phenotypic subtypes of patients with LSDs identified by NBS in China. Of 27 patients, 11.1% had early-onset forms (2 patients with Gaucher disease type II and 1 patient with classic Fabry disease) and nearly 90% (88.9%) had later-onset forms (9 patients with Krabbe, 7 patients with Fabry, 5 patients with ASMD type B, and 3 patients with Pompe disease). Optimum treatments were provided to patients with LSDs, and proper genetic counseling was provided to their families.

### Limitations

This study has several limitations. First, newborns who screened positive with GAA activity between 10% and 20% of the daily median did not receive genetic testing, which may have missed some patients who were positive. However, this may be a minor limitation because pseudodeficiency alleles are common in the Chinese population, affecting the positive rate of NBS for Pompe disease. A new second-tier marker, the creatine/creatinine to GAA ratio, offered a promising approach to improve the specificity of NBS for Pompe disease.^36^ Second, we need a very long time to follow up patients with later-onset disease because of the uncertain age of onset. However, we informed them of the importance of follow-up and initiation of treatment, although they may develop the disease in adulthood. Third, the 1-year screening time is a limitation, and a long period of data analysis will be important to determine the most accurate birth prevalence.

## Conclusions

In this cohort study, we identified a high combined birth prevalence (1 in 1856 births) of LSDs in Shanghai. Enzyme activity measurement was used for the primary screening, followed by molecular genetics and biomarkers confirmatory testing. Clinical assessments assisted in the determination of clinical forms of the 6 LSDs screened. Most live-born infants with Krabbe, Fabry, ASMD type B, or Pompe diseases identified by NBS presented mild clinical manifestations. However, infants with Gaucher disease had severe disease. The main pathogenic variants were c.1448T>C (p.Leu483Pro) in the *GBA* gene, c.995C>G (p.Pro332Arg) in the *SMPD1* gene, c.1901T>C (p.Leu634Ser) in the *GALC* gene, and c.2238G>C (p.Trp746Cys) in the *GAA* gene, contributing to the high prevalence of Gaucher, ASMD, Krabbe, and Pompe diseases in Shanghai. Potential benefits of genetic counseling and benefits of early treatment once enrolled in surveillance programs of live-born children who were identified support the public health role of NBS for LSDs in the general population.

## Notes

Supplement 1. **eTable 1.** The parameters of liquid phase and QSight 210 MD mass spectrometer **eTable 2.** Age at onset, age at diagnosis, and clinical phenotypes of reported patients with the variants of Fabry disease **eTable 3.** The birth prevalences of 6 LSDs in different regions
eReferences.

Supplement 2.
Data Sharing Statement
