# Errors in Units in Methods and Measure Type in Results

**DOI:** 10.1001/jamanetworkopen.2024.19382

**Published:** 2024-05-30

In the Original Investigation titled “Newborn Screening for 6 Lysosomal Storage Disorders in China,”^1^ published May 13, 2024, units for the lysosomal enzyme activity assay were incorrectly listed as uL instead of μL in the Methods and a range for median age was incorrectly reported as an IQR in the Results. The article has been corrected.^1^
