# Supplementary material for: Newborn Screening for 6 Lysosomal Storage Disorders in China
Source: JAMA Netw Open. 2024 May 13;7(5):e2410754. doi: 10.1001/jamanetworkopen.2024.10754 (PMC11091758; doi:10.1001/jamanetworkopen.2024.10754)
Supplement: Supplement 1. — eTable 1. The parameters of liquid phase and QSight 210 MD mass spectrometer eTable 2. Age at onset, age at diagnosis, and clinical phenotypes of reported patients with the variants of Fabry disease eTable 3. The birth prevalences of 6 LSDs in different regions eReferences. [file jamanetwopen-e2410754-s001.pdf]

Supplemental Online Content

Chang S, Zhan X, Liu Y, et al. Newborn screening for 6 lysosomal storage disorders in China. *JAMA Netw Open*. 2024;7(5):e2410754.
doi:10.1001/jamanetworkopen.2024.10754

eTable 1. The parameters of liquid phase and QSight 210 MD mass spectrometer
eTable 2. Age at onset, age at diagnosis, and clinical phenotypes of reported patients with the variants of Fabry disease
eTable 3. The birth prevalences of 6 LSDs in different regions
eReferences.

This supplemental material has been provided by the authors to give readers additional information about their work.

© 2024 Chang S et al. *JAMA Network Open.*

eTable 1. The parameters of liquid phase and QSight 210 MD mass spectrometer

Name	MRM (Q1)	MRM (Q3)	DWELL TIME (ms)	EV
ABG	384.30	264.30	40	10
ABG IS	391.40	271.30	40	10
ASM	398.40	264.30	40	10
ASM IS	405.40	264.30	40	10
GALC	412.40	264.30	40	10
GALC IS	417.40	264.30	40	10
IDUA	426.20	317.20	40	10
IDUA IS	431.30	322.20	40	10
GLA	484.30	384.20	40	10
GLA IS	489.30	389.30	40	10
GAA	498.30	398.20	40	10
GAA IS	503.30	403.30	40	10
MS/MS condition				
Source 1 temperature	120 ℃			
HSID temperature	100 ℃			
Drying Gas	100 ℃			
Nebulizer Gas 1	150 psi			
ElectroSpray V1 Pos	5000 V			
Entrance Voltage	30 V			
Collision Cell Lens 2	-120 V			
Collision Energy	-50 V			
LC Conditions				
Mobile Phase	80/20 acetonitrile/water (Fraction A)			
Liquid phase gradient	Fraction A 100%; 0.01~0.30 min,0.15 mL/min; 0.30~0.45 min, 0.50 mL/min; 0.45~0.60 min, 0.15 mL/min.			
Washes	80/20 acetonitrile/water			
Inject Sample Flow Rate	10 uL/s			
Inject volume	10 uL			

eTable 2. Age at onset, age at diagnosis, and clinical phenotypes of reported patients with the variants of Fabry disease.

No	Nucleotide Change	Protein change	Age at onset	Age at diagnosis	Reported clinical phenotype	Reference
1	c.718_719del	p.Lys240Glufs*9	5years	22years	Classical phenotype	1
2	c.640-801G>A	/	60years	38-64years	Later-onset cardiac phenotype	2, 3
3	**c.428C>T**	**p.Ala143Val**	/	3days	Later-onset phenotype	This study
4	c.358C>G	p.Leu120Val	/	3days	Later-onset phenotype	4
5	c.335G>A	p.Arg112His	19-35years	19-37years	Atypical renal-dominant phenotype	1
6	c.1067G>A	p.Arg356Gln	/	3days	Later-onset phenotype	4
7	c.593T>C	p.Ile198Thr	/	27-46years	Later-onset phenotype	5, 6
8	c.137A>C	p.His46Pro	/	5-50years	Later-onset phenotype	4, 7

© 2024 Chang S et al. *JAMA Network Open.*

eTable 3. The birth prevalences of 6 LSDs in different regions.

Prevalence/Region	Gaucher	NPA/B	Krabbe	MPS-I	Fabry	Pompe	Reference
North East Italy	1:22 000	/	/	1:44 000	1:9 000	1:22 000	8
Washington state	1:44 000	1:44 000	/	1:11 000	1 in 10 000 males	1:44 000	9
New York	1:4 000	/	/	/	1:9 000	1:19 000	10
Austria	1:17 000	/	/	/	1:3 800	1:8 700	11
Shandong, China	/	/	1:8 000	/	1:13 000	1:13 000	12
Taiwan, China	1:100 000	/	/	1:30 000	1:3 000	1:12 000	13
Shanghai, China	1:25054	1:10022	1:5568	/	1:6264	1:16702	This study

© 2024 Chang S et al. *JAMA Network Open.*

References

1. Pan X, Ouyang Y, Wang Z, et al. Genotype: A Crucial but Not Unique Factor Affecting the Clinical Phenotypes in Fabry Disease. PLoS One. 2016;11(8):e0161330. doi:10.1371/journal.pone.0161330.
2. Hsu TR, Hung SC, Chang FP, et al. Later Onset Fabry Disease, Cardiac Damage Progress in Silence: Experience With a Highly Prevalent Mutation. J Am Coll Cardiol. 2016;68(23):2554-2563. doi:10.1016/j.jacc.2016.09.943.
3. Kubo T, Ochi Y, Baba Y, et al. Prevalence and clinical features of Fabry disease in Japanese male patients with diagnosis of hypertrophic cardiomyopathy. J Cardiol. 2017; 69(1):302-307. doi:10.1016/j.jjcc.2016.05.014.
4. Hwu WL, Chien YH, Lee NC, et al. Newborn screening for Fabry disease in Taiwan reveals a high incidence of the later-onset GLA mutation c.936+919G>A (IVS4+919G>A). Hum Mutat. 2009;30(10):1397-1405. doi:10.1002/humu.21074.
5. Varela P, Mastroianni Kirsztajn G, Motta FL, et al. Correlation between GLA variants and alpha-Galactosidase A profile in dried blood spot: an observational study in Brazilian patients. Orphanet J Rare Dis. 2020;15(1):30. doi:10.1186/s13023-019-1274-3.
6. Echevarria L, Benistan K, Toussaint A, et al. X-chromosome inactivation in female patients with Fabry disease. Clin Genet. 2016;89(1):44-54. doi:10.1111/cge.12613
7. Tsuboi K, Yamamoto H. Clinical observation of patients with Fabry disease after switching from agalsidase beta (Fabrazyme) to agalsidase alfa (Replagal). Genet Med. 2012; 14(9):779-786. doi:10.1038/gim.2012.81.
8. Burlina AB, Polo G, Salviati L, et al. Newborn screening for lysosomal storage disorders by tandem mass spectrometry in North East Italy. J Inherit Metab Dis. 2018;41(2):209-219. doi:10.1007/s10545-017-0098-3.
9. Elliott S, Buroker N, Cournoyer JJ, et al. Pilot study of newborn screening for six lysosomal storage diseases using Tandem Mass Spectrometry. Mol Genet Metab. 2016;118(4):304-309. doi:10.1016/j.ymgme.2016.05.015.
10. Wasserstein MP, Caggana M, Bailey SM, et al. The New York pilot newborn screening program for Lysosomal storage diseases: Report of the First 65,000 Infants. Genet Med. 2019; 21(3):631-640. doi:10.1038/s41436-018-0129-y.
11. Mechtler TP, Stary S, Metz TF, et al. Neonatal screening for lysosomal storage disorders: feasibility and incidence from a nationwide study in Austria. Lancet. 2012; 379(9813):335-341. doi:10.1016/S0140-6736(11)61266-X.
12. Li R, Tian L, Gao Q, et al. Establishment of Cutoff Values for Newborn Screening of Six Lysosomal Storage Disorders by Tandem Mass Spectrometry. Front Pediatr. 2022;10:814461. doi:10.3389/fped.2022.81446.
13. Liao HC, Chiang CC, Niu DM, et al. Detecting multiple lysosomal storage diseases by tandem mass spectrometry-A national newborn screening program in Taiwan. Clin Chim Acta. 2014;431:80-86. doi: 10.1016/j.cca.2014.01.030

© 2024 Chang S et al. *JAMA Network Open.*
