# Supplementary material for: Newborn Screening for 6 Lysosomal Storage Disorders in China
Source: JAMA Netw Open. 2024 May 13;7(5):e2410754. doi: 10.1001/jamanetworkopen.2024.10754 (PMC11091758; doi:10.1001/jamanetworkopen.2024.10754)
Supplement: Supplement 2. — Data Sharing Statement [file jamanetwopen-e2410754-s002.pdf]

Data Sharing Statement

Chang. Newborn Screening for 6 Lysosomal Storage Disorders in China. *JAMA Netw Open*. Published online May 13, 2024. doi:10.1001/jamanetworkopen.2024.10754

Data
Data available: No
